# Cyclic Alternating Pattern in Obstructive Sleep Apnea Patients with versus without Excessive Sleepiness

**DOI:** 10.1155/2018/8713409

**Published:** 2018-05-16

**Authors:** Selda Korkmaz, Nedime Tugce Bilecenoglu, Murat Aksu, Tahir Kurtulus Yoldas

**Affiliations:** ^1^Department of Neurology, Istanbul Aydin University, Faculty of Medicine, Istanbul, Turkey; ^2^Department of Neurology, Acibadem Kayseri Hospital, Kayseri, Turkey; ^3^Department of Neurology, Acibadem Kayseri Hospital, Acibadem University Faculty of Medicine, Istanbul, Turkey; ^4^Department of Neurology, Ankara Training and Research Hospital, Ankara, Turkey

## Abstract

**Background:**

One of the main hypotheses on the development of daytime sleepiness (ES) is increased arousal in obstructive sleep apnea (OSA). Cyclic alternating pattern (CAP) is considered to be the main expression of sleep microstructure rather than arousal. Therefore, we aimed to investigate whether there is any difference between OSA patients with versus without ES in terms of the parameters of sleep macro- and microstructure and which variables are associated with Epworth Sleepiness Scale (ESS) score.

**Methods:**

Thirty-eight male patients with moderate to severe OSA were divided into two subgroups by having been used to ESS as ES or non-ES.

**Results:**

There was no difference between two groups in clinical characteristics and macrostructure parameters of sleep. However, ES group had significantly higher CAP rate, CAP duration, number of CAP cycles, and duration and rate of the subtypes A2 (*p* = 0.033, 0.019, 0.013, and 0.019, respectively) and lower mean phase B duration (*p* = 0.028) compared with non-ES group. In correlation analysis, ESS score was not correlated with any CAP measure.

**Conclusions:**

OSA patients with ES have increased CAP measures rather than those without ES.

## 1. Introduction

Obstructive sleep apnea (OSA) is the most common sleep related breathing disorder and characterized by complete or partial repetitive interruptions of airflow during sleep. Its prevalence was found 2% in women and 4% in men but, nowadays, possibly higher [1]. Daytime sleepiness (ES) has been considered one of the cardinal symptoms of OSA [2], but the data about the prevalence and underlying pathophysiological mechanism of ES in patients with OSA are controversial. Several studies showed that many OSA patients experience ES whereas the other studies did not [3–7]. This discrepancy can result from differences among studies in study design, statistical methodology, and tools used to evaluate ES. In those studies, many factors including sleep fragmentation, hypoxemia, and obesity have been proposed as main determinants of ES in OSA patients [8–12].

Repetitive transient cortical EEG activities occurring as a response to internal or external stimuli during sleep are a physiologic event, but an enhancement of these activities results in disruption in normal sleep architecture. Although arousal is defined as a marker of sleep disruption, each epoch encompasses several short-timed transient events not meeting diagnostic criteria of arousal. Detection and analysis of them are called cyclic alternating pattern (CAP) and it is considered to be the main expression of sleep microstructure [13]. As a marker of sleep instability, CAP describes a physiological oscillating state involving cerebral activities, autonomic functions, and behavior features. High amounts of CAP rate indicate that one or more factors interfere with sleep consolidation. Several studies have shown that sleep disorders such as OSA, PLM, and insomnia have increased CAP measures [14–16]. Furthermore, this enhancement has been dramatically reduced by CPAP titration in patients with OSA [17].

The aim of present study is to investigate whether there is any difference between OSA patients with and without ES in terms of the parameters of sleep macro- and microstructure and which variables are associated with Epworth Sleepiness Scale (ESS) score in OSA patients with ES. To our knowledge, CAP and its association with ES in OSA patients have not been previously investigated.

## 2. Method

### 2.1. Subjects

The study was conducted at the sleep units of three different centers, approved by the institutional ethical committee, and carried out in accordance with the principles of the Helsinki Declaration.

38 male patients with OSA were retrospectively recruited from among 478 patients who underwent polysomnography (PSG) at the sleep laboratory. OSA was diagnosed according to the International Classification of Sleep Disorders II. The inclusion criteria of the study were (1) age 40 to 60 years; (2) male gender; (3) AHI > 15; (4) PLMI < 5; (5) absence of psychiatric and neurologic disorders such as depression and stroke; (6) absence of another sleep disorder such as narcolepsy, insomnia, and RLS; (7) use of drug affecting the sleep structure. 38 OSA patients meeting inclusion criteria were divided into two subgroups as ES and non-ES. Baseline clinical characteristics and polysomnographic variables indicating macro- and microstructure of sleep of these 38 patients were compared between patients with and without ES. Daytime sleepiness was diagnosed in the presence of an Epworth Sleepiness Scale score (ESS) > 10.

### 2.2. Video Polysomnography

A full night PSG recording was performed by using a computerized recording system (Embla® RemLogic™) consisting of (1) sleep stage scoring through six channel electroencephalography (EEG) (F4-M1, C4-M1, O4-M1, F3-M2, C3-M2, O3-M2), two channel electrooculography (EOG), and one channel electromyography (EMG); (2) respiratory scoring through a thermistor as well as a nasal pressure sensor for apnea–hypopnea detection, piezo-crystal effort belts for thoraco-abdominal movement detection, and a pulse-oximeter; (3) two-lead electrocardiogram (ECG); and (4) leg movement scoring through bilateral tibial EMG and a body position detector.

### 2.3. Analysis of Sleep Macrostructure

Sleep was manually scored in 30-s epochs according to the criteria of the American Academy of Sleep Medicine (AASM) [18]. Sleep stages were scored as W (wake), N1 (stage 1 sleep), N2 (stage 2 sleep), N3 (stage 3 sleep-SWS), and R (rapid eye movement (REM) sleep). The PSG parameters of sleep macrostructure consisted of the following: (1) sleep scoring data: total sleep time (TST; in minutes), sleep latency (SL; lights out to first epoch of any sleep in minutes), sleep efficiency ([TST/total recording time] × 100), wake after sleep onset (WASO; stage W during total recording time, minus SL, in minutes), duration of each stage, percent of TST in each stage (time in each stage/TST), and stage REM latency (sleep onset to first epoch of stage REM in minutes); (2) respiratory events: apnea-hypopnea index (AHI; total number of apneas and hypopneas × 60/TST), lowest O2 saturation (min SpO_2_), and mean nocturnal oxygen saturation (mean SpO_2_); apnea was defined as a drop in the peak thermal sensor excursion by ≥90% of baseline lasting at least 10 seconds accompanied by respiratory effort movement. Hypopnea was defined as nasal pressure signal excursions drop by ≥30% of baseline with ≥4% desaturation from pre-event baseline, or ≥50% of baseline with ≥3% desaturation from pre-event baseline or the event is associated with arousal, associated with respiratory effort; (3) movement events: periodic leg movements of sleep (PLMS) index (PLMI; number of PLMS × 60/TST) according to the AASM criteria. The data were scored by a sleep medicine specialist who was masked to the status of subjects.

### 2.4. The Analysis of Sleep Microstructure

#### 2.4.1. Arousal Analysis

Arousal was scored according to AASM rules [19]. Arousal was defined as an abrupt shift in EEG frequency, including alpha, theta, and/or frequencies higher than 16 Hz (but not spindles) lasting at least 3 s, with at least 10 s of stable sleep preceding the change. Scoring an arousal in REM sleep mandated an additional increase in chin EMG tone for at least 1 s. Complete awakening from sleep was not counted as arousal. An arousal could be accompanied by an increase in EMG activity, heart rate, and/or body movements.

#### 2.4.2. CAP Analysis

In the present study, CAP was scored according to currently accepted criteria [20]. CAP is described as a periodic EEG activity occurring during NREM sleep and characterized by repeated sequences of transient events which recurs at intervals up to 2 minutes. These sequences are composed of a succession of CAP cycles comprising a phase A and the following phase B which is the background EEG activity separating two consecutive phases A. Phase A activities can be classified into three subtypes. Subtype A1 is predominantly composed of slow waves while subtype A3 contains fast EEG activities. Subtype A2 is a combination of both.

**Figure 1:**
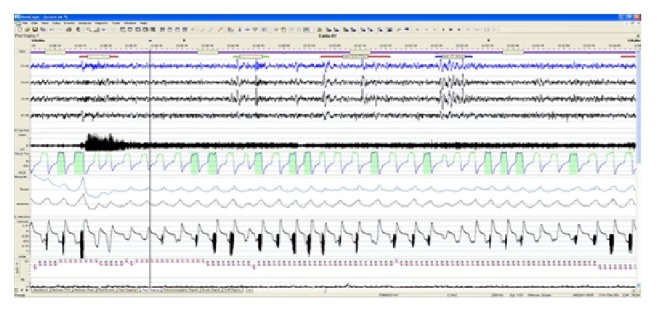
A sample of CAP sequence in the study group.

All CAP sequences begin with a phase A and end with a phase B. Each phase of CAP may vary from 2 to 60 s in duration. Accordingly, a phase A is scored within a CAP sequence only if it precedes and/or is followed by another phase A in the temporal range of 2−60 s. If there were three consecutive A phases followed by a non-CAP condition, the CAP sequence was stopped at the end of the second B-phase and the third A phase A was quantified as non-CAP. A continuous NREM sleep EEG pattern without any phase A for more than 60 s is scored as non-CAP. Isolated A phases, not tied into CAP sequences, were also included in non-CAP periods. Based on these criteria, a sample of CAP sequence scored in the current study was presented in Figure 1.

The following CAP variables were measured in the present study: CAP rate (percentage of total NREM sleep time occupied by CAP sequences); percentage and duration of each A phase subtype; A1, A2, and A3 index; number of phases A1, A2, or A3 per hour of NREM sleep; number and duration of CAP cycle; and number and duration of B phases. All these variables were scored manually by the sleep specialist blinded to subject identity.

### 2.5. Epworth Sleepiness Scale (ESS)

The ESS is an eight-item questionnaire that is used to assess the severity of daytime sleepiness in various situations [21]. The patient is required to rate his or her likelihood of sleepiness from 0 to 3 for each question. The items are as follows: (1) sitting and reading; (2) watching television; (3) sitting inactive in a public place (e.g., a theater or a meeting); (4) as a passenger in a car for an hour without a break; (5) lying down to rest in the afternoon when circumstances permit; (6) sitting and talking to someone; (7) sitting quietly after lunch without alcohol; and (8) in a car, while stopped for a few minutes in traffic. The total ESS score ranges from 0 to 24.

The patient group was divided into two subgroups as ES, which had a score of higher than 10, or non-ES, which had a score of equal to or lower than 10.

### 2.6. Statistics

All variables were examined by normality test prior to analysis. Normally distributed variables (including age, height, TST, WASO, the duration and rate of N2, N3, and REM, the number and index of arousal, CAP rate, mean A and B phases, the duration and rate of A1 subtype, the rate of A2 and A3 subtypes, and A2 and A3 index) were expressed as mean ± standard deviation and analyzed by two independent Student's *t*-tests for examining the differences of means of different variables between the two patient subgroups (ES and non-ES). Then, skewed data (including weight, BMI, ESS score, SE, SL, the duration and rate of N1, the mean and lowest O_2_ saturation, AHI, CAP duration, the number of CAP cycles, the duration of A2 and A3 subtypes, and A1 index) were expressed as median (interquartile range), and Mann–Whitney *U* nonparametric test was applied to examine differences between groups. Correlation analyses were performed using Spearman's correlation test. A *p* value less than 0.05 was accepted as statistically significant.

## 3. Results

Baseline clinical characteristics and ESS score of study group are presented in Table 1. There was no significant difference between groups in age, height, weight, and BMI. ESS score was significantly higher in ES group than non-ES group (*p*≲0001).

There was no difference between groups in the macrostructural parameters of sleep. The comparison of the parameters of sleep macrostructure of patients with and without ES was summarized in Table 2. Regarding sleep microstructure parameters, there was no difference between groups in the number of arousal, arousal index, mean phase A duration, and the duration and rate of the subtypes A1 and A3. However, CAP rate, CAP duration, the number of CAP cycles, and the duration and rate of the subtypes A2 were found significantly higher in ES group than non-ES group (*p* = 0.033, 0.019, 0.013, and 0.019, respectively). Conversely, mean phase B duration was lower in ES group than non-ES group (*p* = 0.028). All parameters of sleep microstructure were summarized in Table 3.

**Figure 2:**
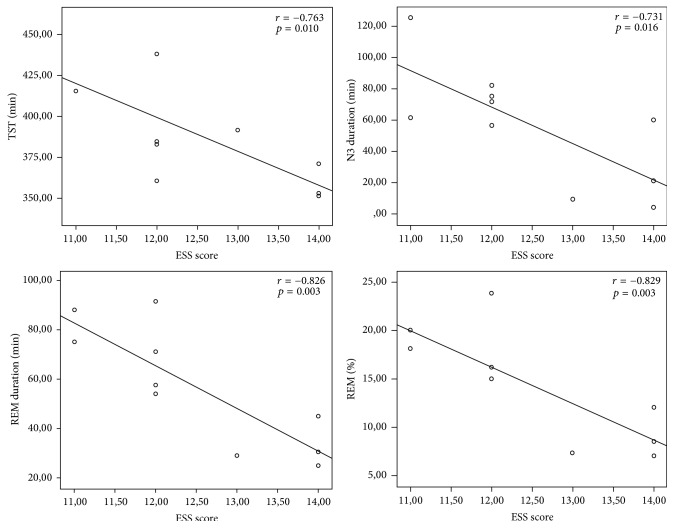
Associations between ESS score and TST and N3 duration and REM duration and REM rate.

**Figure 3:**
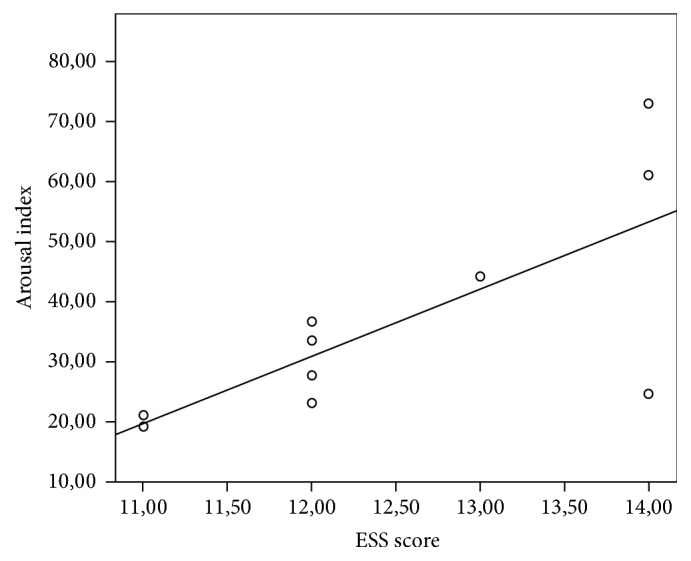
Relationship between ESS score and arousal index.

In correlation analysis, ESS score was significantly negatively correlated with TST, N3 duration, and the duration and rate of REM in ES group (*r* = −0.763, −0.731, −0.826, −0.275 and −0.829; *p* = 0.01, 0.016, 0.003, and 0.003, respectively) whereas ESS score was significantly positively correlated with arousal index (*r* = 0.756, *p* = 0.011) (Figures 2 and 3). Nevertheless, there was no correlation between ESS score and the parameters of CAP. Spearman's Correlation Coefficients between ESS score and the parameters of sleep architecture in ES group were presented in Table 4. Any correlation was not observed in non-ES group.

## 4. Discussion

Up to now, several studies proposed that ES is associated with some clinical parameters and the variables of conventional PSG [6, 7, 10, 11, 22–24] whereas other studies did not confirm these data [8, 25, 26]. In these studies, OSA patients with ES have had higher AHI, TST, arousal index, and BMI and lower oxygen saturation, but lower N3 and REM duration compared to those without ES. We did not observe any difference between two groups in terms of the parameters of sleep macrostructure defined by conventional PSG scoring and clinical characteristics similar to several previous studies. As a result, ES is not explained by only clinical and/or conventional PSG parameters.

Respiratory events in OSA show a cyclic pattern as a cessation and resumption of breathing for certain duration, and the resumption of breathing is associated with an arousal from sleep. General agreement on arousal is to be an indicator of sleep fragmentation [27]. However, periodic EEG activities during sleep may remain unexplored if standard scoring criteria exclusively are used for arousal. Therefore, CAP which is an alternative scoring system for arousal and concomitant physiologic events during sleep. Current studies have focused on sleep instability and its association with CAP. No studies have previously investigated CAP in OSA patients with versus without ES. However, only one study has demonstrated a positive correlation between CAP rate and ESS in patients with upper airway resistance syndrome as well as a reduced NREM sleep and an elevated CAP rate [28]. Our study revealed that OSA patients with ES had an elevation in CAP measures including CAP rate and duration, the number of CAP cycle, the duration and rate of subtype A2, and reduction in mean phase B compared to ones without ES whereas arousal index was not found different between groups. The finding that OSA patients with ES have increased CAP is consistent with literature data suggesting that more disruption of sleep continuity is associated with more daytime sleepiness [14]. Based on the results of current study and previous data, further studies are needed to clarify the association between CAP and ES. When a direction of relationship between ES and CAP is revealed, CAP can be used a target in the therapy of ES or a following parameter for OSA patients with ES.

The reason why subtype A2 is higher than A1 and A3 is not explained in the present study. Additionally, decreased mean phase B in ES group is contradicting with previous studies reporting that mean phase B is increased in OSA patients and in a temporal relationship with respiratory events [14]. In the present study, the reduction in mean phase B seems to be protective to sleep fragmentation.

On the other hand, in correlation analysis, we found that ESS score was negatively correlated with TST, N3 duration, and the duration and rate of REM and positively correlated with arousal index in ES group whereas there was no correlation between CAP measures and ES. Additionally, any significant correlation was not observed in non ES group.

Several limitations should be considered when assessing the results of present study. First, the study sample size is quite small because of our strict inclusion criteria. Second, study groups were formed based on their ESS score. ESS reflects to individual's sleepiness subjectively and could lead to over- and underestimation of ES. However, it offers advantages in terms of low cost and easy application, although MSLT is an objective neurophysiologic test measuring sleepiness. Additionally, some studies have reported that ESS has a better or equal ability to MSLT in the definition of ES [29]. Lastly, present study does not include healthy control group. The study involving healthy controls as well as OSA patients with and without ES will obtain further information about discriminating features among groups.

As a result, this present study showed that amount of CAP was increased in OSA patients with ES versus without ES. We do not know the clinical importance of the elevation of CAP in ES group. Moreover, any correlation was not found between CAP and ES. Based on the previous and the present study results, ES is a multifactorial condition and not explained by only clinical or PSG parameters including arousal, CAP. Because increased CAP rate in OSA patients with ES has been firstly documented in the present study, firstly, this finding should be confirmed by the future studies and then its clinical consequence should be investigated.

## Figures and Tables

**Table 1:** Comparison of clinical characteristics and ESS score in OSA patients with and without ES (ES versus non-ES).

	ES (*n* = 10)	Non-ES (*n* = 28)	*p* value
Age (years)	55.55 (4.43)	53.94 (4.52)	0.713
Weight (kg)	87.5 (14.5)	96.5 (21.68)	0.205
Height (cm)	175.8 (7.74)	178.86 (7.41)	0.276
BMI (kg/m^2^)	28.7 (2.77)	28.85 (7.53)	0.757
ESS score	12 (2.25)	7.5 (4.75)	**<0001**

**Table 2:** Comparison of parameters of sleep macrostructure in OSA patients with and without ES (ES versus non-ES).

	ES (*n* = 10)	Non-ES (*n* = 28)	*p* value
TST, min	388.94 (32.86)	365.66 (77)	0.202
SE	74.1 (2.17)	74.5 (17.42)	0.708
SL	12 (15.88)	9 (8.9)	0.660
WASO	130.48 (24.57)	126.56 (65.97)	0.791
N1 duration, min	62 (23)	69 (62.25)	0.883
N1, (%)	16.8 (4.5)	20.55 (15.13)	0.590
N2 duration, min	196.94 (47.43)	199.39 (57.82)	0.905
N2, (%)	50.62 (12.04)	53.97 (8.18)	0.335
N3 duration, min	56.75 (36.67)	42.27 (31.4)	0.239
N3, (%)	14.36 (8.97)	11.42 (8.21)	0.349
REM duration, min	56.65 (24.34)	45.68 (22.27)	0.200
REM, (%)	14.33 (5.56)	12.31 (5.48)	0.326
Mean O_2_ sat	94 (2.55)	93.5 (2.17)	0.257
Lowest O_2_ sat	84 (5)	80.5 (11.75)	0.503
AHI	34.85 (46.35)	32.8 (32)	0.757

**Table 3:** Comparison of parameters of sleep microstructure in OSA patients with and without ES (ES versus non-ES).

	ES (*n* = 10)	Non-ES (*n* = 28)	*p* value
The number of arousal	216.2 (73.25)	207.5 (117.19)	0.828
Arousal index	36.4 (18.04)	35.05 (16.85)	0.834
CAP duration, min	212.15 (70.88)	162.9 (99.33)	**0.019**
CAP rate (%) = CAP (min)/NREM (min) × 100	65.98 (11.53)	53.93 (15.72)	**0.033**
The number of CAP cycles	375.5 (165.5)	293 (130.25)	**0.013**
Mean phase A duration, min	16.79 (1.72)	16.12 (2.11)	0.373
Mean phase B duration, min	17.41 (2.73)	20.22 (3.49)	**0.028**
Subtype A1 duration, min	29.53 (26.76)	21.25 (19.17)	0.299
Subtype A1 (%)	8.93 (7.87)	6.38 (5.39)	0.265
Subtype A1 index (The number of A1/NREM hours)	8.6 (7.93)	3.5 (8.4)	0.070
Subtype A2 duration, min	49.35 (33.1)	21.9 (25.7)	**0.019**
Subtype A2 (%)	15.17 (6.35)	9.69 (6.98)	**0.036**
Subtype A2 index (The number of A2/NREM hours)	13.95 (9.82)	12.49 (8.62)	0.661
Subtype A3 duration, min	145.95 (40.1)	113.05 (38)	0.051
Subtype A3 (%)	41.9 (6.02)	37.85 (11.4)	0.169
Subtype A3 index (The number of A3/NREM hours)	38.51 (13.83)	41.56 (11.66)	0.503

**Table 4:** Spearman's Correlation Coefficients between ESS score and the parameters of sleep architecture in ES group.

	ES (*n* = 10)	Non-ES (*n* = 28)	*p* value
The number of arousal	216.2 (73.25)	207.5 (117.19)	0.828
Arousal index	36.4 (18.04)	35.05 (16.85)	0.834
CAP duration, min	212.15 (70.88)	162.9 (99.33)	**0.019**
CAP rate (%) = CAP (min)/NREM (min) × 100	65.98 (11.53)	53.93 (15.72)	**0.033**
The number of CAP cycles	375.5 (165.5)	293 (130.25)	**0.013**
Mean phase A duration, min	16.79 (1.72)	16.12 (2.11)	0.373
Mean phase B duration, min	17.41 (2.73)	20.22 (3.49)	**0.028**
Subtype A1 duration, min	29.53 (26.76)	21.25 (19.17)	0.299
Subtype A1 (%)	8.93 (7.87)	6.38 (5.39)	0.265
Subtype A1 index (The number of A1/NREM hours)	8.6 (7.93)	3.5 (8.4)	0.070
Subtype A2 duration, min	49.35 (33.1)	21.9 (25.7)	**0.019**
Subtype A2 (%)	15.17 (6.35)	9.69 (6.98)	**0.036**
Subtype A2 index (The number of A2/NREM hours)	13.95 (9.82)	12.49 (8.62)	0.661
Subtype A3 duration, min	145.95 (40.1)	113.05 (38)	0.051
Subtype A3 (%)	41.9 (6.02)	37.85 (11.4)	0.169
Subtype A3 index (The number of A3/NREM hours)	38.51 (13.83)	41.56 (11.66)	0.503

## Data Availability

The data of study subjects were retrospectively obtained from recordings at sleep laboratory.
